# A Call to Action: Reinvigorating Interest and Investments in Health Infrastructure

**DOI:** 10.9745/GHSP-D-21-00674

**Published:** 2021-12-31

**Authors:** Lindsay M. Mallick, Joshua Amo-Adjei

**Affiliations:** a Department of Family Science, Maternal and Child Health Program, School of Public Health, University of Maryland, College Park, MD, USA.; b Maryland Population Research Center, College Park, MD, USA.; c Avenir Health, Glastonbury, CT, USA.; d University of Cape Coast, Cape Coast, Ghana.

## Abstract

Infrastructure investments can contribute substantially to alleviating burdens of morbidity and mortality while also providing a positive return on investment in the long term.

See related article by Rokicki et al.

Person-centered care and measurement of the client experience has captured the spotlight in the field of quality of care,^1^ especially amidst growing awareness of pervasive mistreatment and disrespectful care.^2^^,^^3^ Despite the importance of this topic, a focus on strengthening infrastructure cannot fall by the wayside. In this issue of *GHSP*, Rokicki et al. draw our attention to structural aspects of delivery care and demonstrate their importance in health care delivery.^4^

In essence, infrastructure is essential for high-quality care, yet improvements in health infrastructure in low- and middle-income countries (LMICs) have not progressed on pace with the increasing demand for health services, especially in sub-Saharan Africa.^5^ A renewed focus on infrastructure to both improve health and protect and retain the health care workforce—a focus that works in tandem with efforts to promote person-centered care, is environmentally friendly, and aims to equip facilities to handle infectious disease outbreaks—is direly needed. Here, we make the plea to reinvigorate interest and investments in health infrastructure.

## QUALITY CANNOT OCCUR IN THE ABSENCE OF ADEQUATE INFRASTRUCTURE

In the past several decades, we have seen a dramatic increase in the use of health care facilities, especially for childbirth in LMICs, but the investments in improving access and use of services have not yielded expected decreases in maternal and newborn deaths.^6^^,^^7^ As Rokicki et al. mention,^4^ lack of high-quality care for many common, treatable conditions currently contributes to more excess deaths than lack of use of health care facilities,^8^ thus warranting further examination of health systems and quality of care.

Quality of care is a multidimensional construct that encompasses a spectrum of inputs and processes integral for achieving optimal health and satisfaction with care. Infrastructure—namely, electricity, water, equipment, and physical resources—along with medicines and human resources, is the most basic, foundational requirement of quality of care. These elements, which together constitute readiness for care,^9^ are “nonnegotiables” for providing health services; protecting clients and providers; and avoiding preventable infections, illness, and death.^10^ The Figure depicts a hierarchical structure in which we envision the cross-section of various quality of care frameworks,^11^^–^^14^ wherein infrastructure is at the base of the pyramid of quality health care upon which other aspects of quality and optimal health outcomes rest.

**FIGURE:**
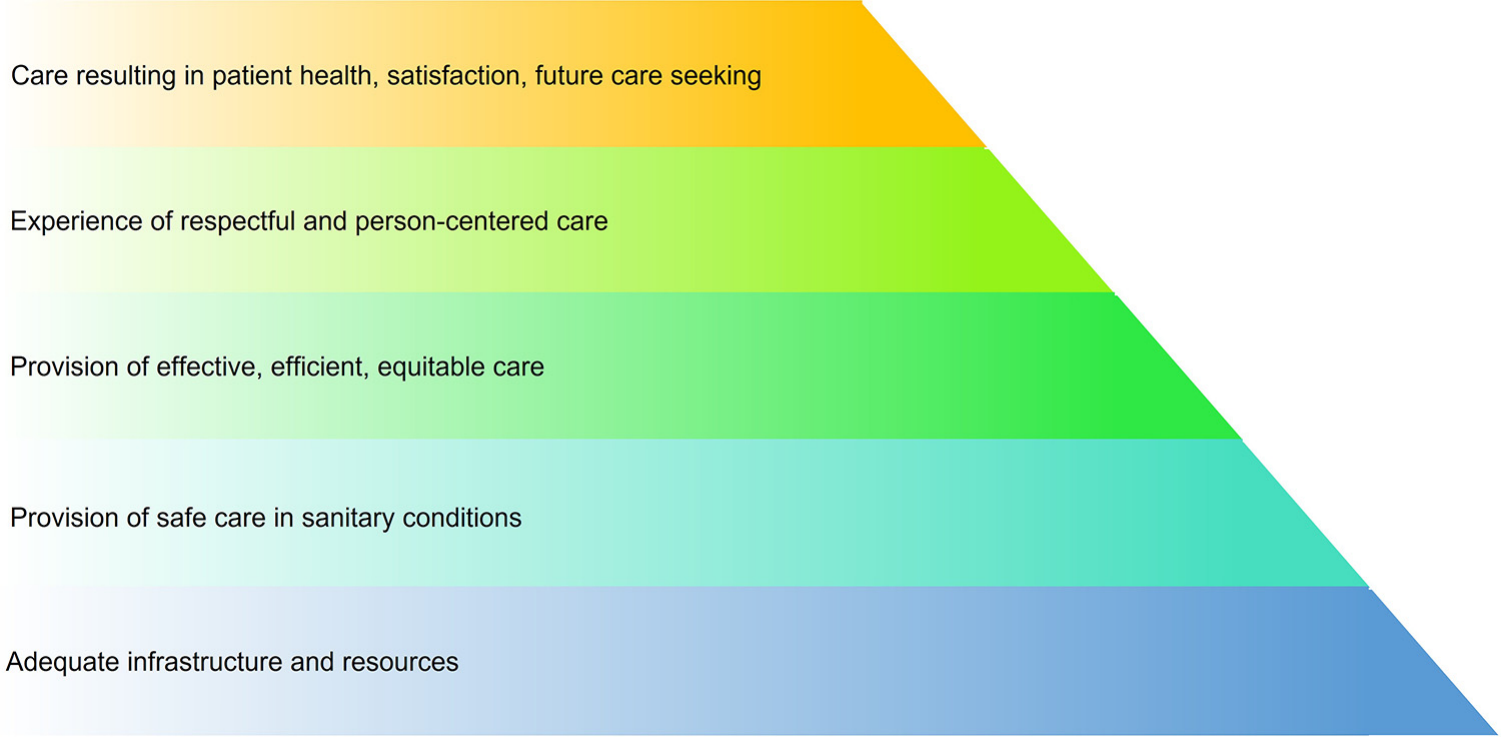
Hierarchy of Quality of Care^a^ ^a^This figure depicts an integration of components of quality of care based on various frameworks, ^11^^–^^14^ organized into a hierarchical structure.

Infrastructure—namely, electricity, water, equipment, and physical resources—along with medicines and human resources, is the most basic, foundational requirement of quality of care, together constituting readiness for care.

While infrastructure is critical for high-quality care, it is not a measure of services provided,^14^ and inadequate service provision can occur even amidst sufficient infrastructure and resources.^15^ Yet, the inverse does not hold, and this cannot be overemphasized. Care cannot be safe without clean water and access to sanitation, it cannot be effective without light and necessary equipment, equitable when rural facilities are most poorly prepared, timely without nearby lifesaving surgical theaters or transportation to access them, and patient-centered without attentive and skilled providers.

## INFRASTRUCTURE IS A REQUIREMENT FOR ADEQUATE PERSON-CENTERED CARE

Infrastructure that allows for adherence to medical standards is a requirement for person-centered care. Person-centered care entails establishing (adult) clients as partners in their care, and through that, trust in the health care system. But without reliable energy, adequate water, and sanitation, and other vital components of health system infrastructure, clients' health and trust are in jeopardy. When these aspects of care are unavailable, it poses a risk to health, further undermines trust, deters future care seeking, and worsens health outcomes.^10^

## HUMAN RESOURCE PROTECTION AND RETENTION THROUGH INFRASTRUCTURE INVESTMENT

Even more important when considering the deleterious impact of the coronavirus disease (COVID-19) pandemic on human resources, adequate infrastructure protects health care workers and is associated with provider satisfaction, recruitment, and retention of the workforce.^16^^–^^18^ Water, sanitation, ventilation, and sharps (e.g., needles) control are necessary for infection prevention and control. Health care workers stationed at facilities without sufficient infrastructure are risking their own health to care for communities and provide suboptimal care for their patients. The lack of infrastructure, most pronounced in rural communities, is a reason often cited for poor retention of health care workers,^16^^–^^18^ especially when their services can be provided more lucratively in environments safer for both clients and staff. Thus, where human resources are sparse, basic infrastructure must be prioritized to acquire, protect, and retain staff.

Where human resources are sparse, basic infrastructure must be prioritized to acquire, protect, and retain staff.

## ADEQUATE ENERGY, WATER, HYGIENE, AND SANITATION WILL SAVE LIVES

With high-quality care and appropriate labor and delivery management, which is contingent on having adequate health infrastructure, an estimated 1.3 million fetal, neonatal, and maternal lives can be saved by 2030 in the 75 countries where nearly all respective deaths occur.^19^ Rokicki et al.^4^ demonstrated that solar electric installation was associated with certain improvements in quality of care, specifically related to infection control, postpartum hemorrhage prevention, and newborn care, which are keystones for mortality prevention.

Electricity, either renewable or grid-powered, is also vital for lighting, medical equipment, refrigeration for preserving medicines and vaccines, ventilation, heating for sterilization, air, or water, and communication systems for record keeping, reporting, and referral processes,^20^^,^^21^ yet more than half of health facilities among 78 African, Asian, and Oceanic LMICs lack access to reliable electricity.^22^ When electricity is available, it is often unreliable, as found in 60% of the facilities in the 78 countries studied. Outages can also be chronic and enduring; for example, Ghana experienced a multiyear power crisis between 2012 and 2016, where electricity was unstable throughout the entire country. These frequent and prolonged power outages were detrimental for the health care system; one study found that the risk of mortality in facilities in Ghana increased by 43% when power was out for more than 2 hours.^23^

There are risks and consequences of seeking care in facilities without adequate water, sanitation, and hygiene (WASH) structures, yet the statistics for the availability of WASH are grimmer than those statistics for electricity. In those same 78 LMICs, only two-thirds of health facilities had improved toilets, 3 of 5 had soap, and half had piped water; worse, only 2% had all 4: toilets, soap, water, and waste management.^22^ The World Health Organization (WHO)/United Nations Children's Fund (UNICEF) Joint Monitoring Programme for WASH estimates that this translates to 900 million people accessing health care facilities without water and 1.5 billion without sanitation services.^24^ Although hospital or facility-acquired infections during birth are largely preventable through tetanus vaccination and clean birth practices (e.g., handwashing and sanitary umbilical cord care), infection acquired during this time is a leading cause of death for mothers and babies.^25^^,^^26^ There is also an increased risk of developing antimicrobial resistance when clients are exposed to human waste (which carries antimicrobial-resistant pathogens) in health care facilities that lack improved toilets or have poor sanitation systems.^24^

## INFRASTRUCTURE NEEDS DURING COVID-19

The COVID-19 pandemic has revealed additional health care system gaps that are essential for managing infectious respiratory diseases such as severe acute respiratory syndrome coronavirus 2 (SARS-CoV-2). Recently, the Demographic and Health Surveys Program highlighted limited readiness to manage infectious diseases according to Service Provision Assessment surveys conducted in health facilities across 3 regions of the Global South. Between 1 to 6 in 10 facilities have communication equipment (a landline, facility-owned, or supported private cellular phone or a short-wave radio)^27^ necessary to refer patients to higher-level facilities for intensive or specialist care and coordinating supportive therapy with oxygen. Although a lack of data is a barrier to assessing oxygen needs more broadly,^28^ only 16% or less of facilities in 7 countries with recent Service Provision Assessment surveys reported availability of oxygen.^27^ However, efforts to scale up oxygen are underway,^28^ and the WHO COVID-19 Essential Supplies Forecasting Tool (https://apps.who.int/iris/rest/bitstreams/1342089/retrieve) can help to estimate needs for oxygen and other equipment, supplies, and drugs for care and treatment of COVID-19. In addition, recommendations for managing patient oxygen needs in resource-constrained settings are compiled and summarized by Serpa Neto et al.^29^

For reducing transmission in health care facilities, adequate air filtration and ventilation are paramount^30^^,^^31^ alongside management of patient flow and personal protective equipment for health care workers. The increased use of intensive care units in the face of COVID-19 further stresses the need for health infrastructure. In intensive care units especially, adequate heating, ventilation, and air conditioning systems are central to maintaining positive pressure or negative pressure rooms to reduce the spread of airborne pathogens.^32^ High-efficiency particulate air filtration or negative pressure zones can reduce the risk of spread of infections in intensive care units, operating rooms, or other areas where procedures generate aerosolized particles.^32^^,^^33^ In resource-constrained settings, these systems are limited given their requirement for stable electricity; however, health care administrators can also use several strategies to limit spread including using personal protective equipment, triaging patients to evaluate urgency, postponing nonemergent procedures, screening and testing for COVID-19, and isolating COVID-19 positive or suspected cases.^34^^,^^35^ These recommendations and others are detailed in the WHO's *Maintaining Essential Health Services: Operational Guidance for the COVID-19 Context*.^35^

As shown by Our World in Data (https://ourworldindata.org/covid-vaccinations), as of December 2021, only 6% of the population in low-income countries had been vaccinated, compared with 74% in high-income countries. The disparate rollout of vaccines against SARS-CoV-2 relates both to vaccine equity—the sharing of vaccines and technology—as well as infrastructure-related challenges in the vaccine cold chain, wherein vaccines must be maintained below or near-freezing temperatures (with temperature variation across different vaccines) from manufacturer to distribution sites.^36^ This is particularly problematic in areas that are rural, lack road infrastructure, are susceptible to transportation barriers during wet seasons, or have facilities that lack electricity for storage.^36^ In 2016, Gavi, the Vaccine Alliance (the organization largely responsible for COVID-19 vaccine rollout in LMICs through COVAX), established the Cold Chain Equipment Optimization Platform, which can be used to plan investments to ensure maintenance of cold chains and, ultimately, improve coverage.^37^ A scale-up of these cold chain management and other vaccine efforts are essential to reduce the burden of COVID; without these, COVID-19 will likely remain endemic,^36^ with a persisting cycle of mutations of new variants.

## CLIMATE-FRIENDLY AND CLIMATE-RESISTANT ENERGY, WATER, AND SANITATION SOLUTIONS

It is most responsible to plan for improvements in infrastructure by considering their environmental impacts. Further, the reality of climate change is such that new installations must be robust to endure extreme and worsening weather conditions. While these installations may require substantial initial investments, they have the benefit of lowering long-term operation costs, reducing health care costs, and yielding an overall cost savings.^10^^,^^38^^,^^39^

The reality of climate change is such that improvements in infrastructure must be robust to endure extreme and worsening weather conditions.

How countries should invest in energy should be determined based on available resources and careful examination of the feasibility and cost-effectiveness of renewable energy versus conventional, grid-powered energy. Renewable or hybrid renewable systems that draw from multiple sources of renewable energy, including wind and solar energy, can deliver sustainable, reliable, environmentally friendly methods of powering basic equipment at health care facilities and have been proposed to meet demands in both urban and rural areas.^21^^,^^40^ The Hybrid Optimization Model for Electric Renewables software (owned by UL), for example, can assist with planning such investments through techno-economic simulations that take into account geographic-specific information.^21^^,^^40^

To improve WASH in health care facilities, the WHO outlines 8 practical steps, including conducting assessments, establishing goals and standards, investing in infrastructure, monitoring WASH systems, training health care workers, engaging communities, and conducting further research.^41^ Recently, UNICEF has supported using wind and solar-powered water pumping systems, that while requiring substantial up-front costs, are environmentally friendly, climate-resistant, and have long-term cost payoffs.^39^ UNICEF details programmatic approaches for improving access to water, including collaboration with climate-sector actors and consideration of local needs and resources.^38^

Among other climate-friendly suggestions for waste management, the WHO recommends consideration of steam-based methods over incineration for decontamination of infectious waste to prevent any further environmental harm.^10^ Moat and Lavis provide an additional resource for developing evidence-informed policies that can be broadly applied to consider other aspects of infrastructure development.^42^

## INFRASTRUCTURE INVESTMENT FOR THE GREATEST GAINS

To be sure, investments in infrastructure for health can be costly and seem untenable in resource-constrained settings. These investments compete with other equally important areas. Nonetheless, infrastructure investments can contribute substantially to alleviating burdens of morbidity and mortality while also providing a positive return on investment in the long term.^10^

In summary, advocacy for better infrastructure must continue until facilities are equipped to handle the health issues they face. Although providing poor quality of care pervades even the most well-equipped service environments, high-quality care is only possible in settings where key infrastructure requirements are met. Any new infrastructure investments should consider environmentally friendly options durable to the extreme weather conditions resulting from climate change, while also bolstering readiness requirements for current and future infectious disease outbreaks. Future human resources, client care-seeking behaviors, and optimal health outcomes depend on these investments.

## Notes

**First published online:** December 9, 2021.
